# Immunotherapy-associated bullous and oral erosive lichenoid eruption successfully treated with hydroxychloroquine

**DOI:** 10.1016/j.jdcr.2024.11.010

**Published:** 2024-11-24

**Authors:** Nadean F. Alnajjar, Steven R. Tahan, Christopher Iriarte

**Affiliations:** a Harvard Medical School, Boston, Massachusetts; b Department of Dermatology, Beth Israel Deaconess Medical Center, Boston, Massachusetts; c Department of Pathology, Beth Israel Deaconess Medical Center, Boston, Massachusetts; d Department of Pathology, Harvard Medical School, Boston, Massachusetts; e Department of Dermatology, Harvard Medical School, Boston, Massachusetts

**Keywords:** cutaneous toxicity, hydroxychloroquine, immune checkpoint inhibitors, immune-related adverse events, immunotherapy, lichen planus, lichenoid dermatitis, lichenoid eruption, pembrolizumab

## Introduction

The advent of immune checkpoint inhibitors (ICIs) has significantly advanced cancer care by enhancing the immune system’s capability to activate an antitumor response. Currently, ICIs have been approved for the treatment of over 20 malignancies with numerous ongoing clinical trials exploring additional therapeutic benefits.^1^ However, ICIs commonly result in cutaneous immune-related adverse effects, which can occur in 30% to 50% of patients.^2^ The clinical manifestations of cutaneous immune-related adverse effects are highly variable, therefore proper classification and management of these toxicities is paramount to avoid disruptions in cancer therapy. Lichenoid eruptions are one such well-described sequelae of ICI therapy that are particularly associated with programmed cell death protein 1/programmed death-ligand 1 inhibitors, and have an incidence of approximately 20% among patients undergoing treatment with anti-programmed cell death protein 1 agents.^2^ The exact etiology of these eruptions remains unclear, but they are hypothesized to result from overactivation of CD8^+^ T cells, leading to inflammation and apoptosis of basal keratinocytes in the epidermis.^3^ Although there are currently no US Food and Drug Administration-approved therapies for ICI-associated lichenoid eruptions, systemic corticosteroids are widely used as first-line agents. Corticosteroids have numerous chronic potential side effects and can blunt the antitumor response of ICIs.^1^ Other commonly employed therapies include acitretin and methotrexate; however, these are often limited by adverse effect profiles.^3^ The increasing use of ICIs warrants the exploration of therapeutic options for long-term control of lichenoid eruptions to ensure that cancer treatment continues uninterrupted. Herein, we present a case of bullous and oral erosive lichenoid eruption induced by pembrolizumab. Our patient was successfully treated with maintenance hydroxychloroquine.

## Case

**Fig 1:**
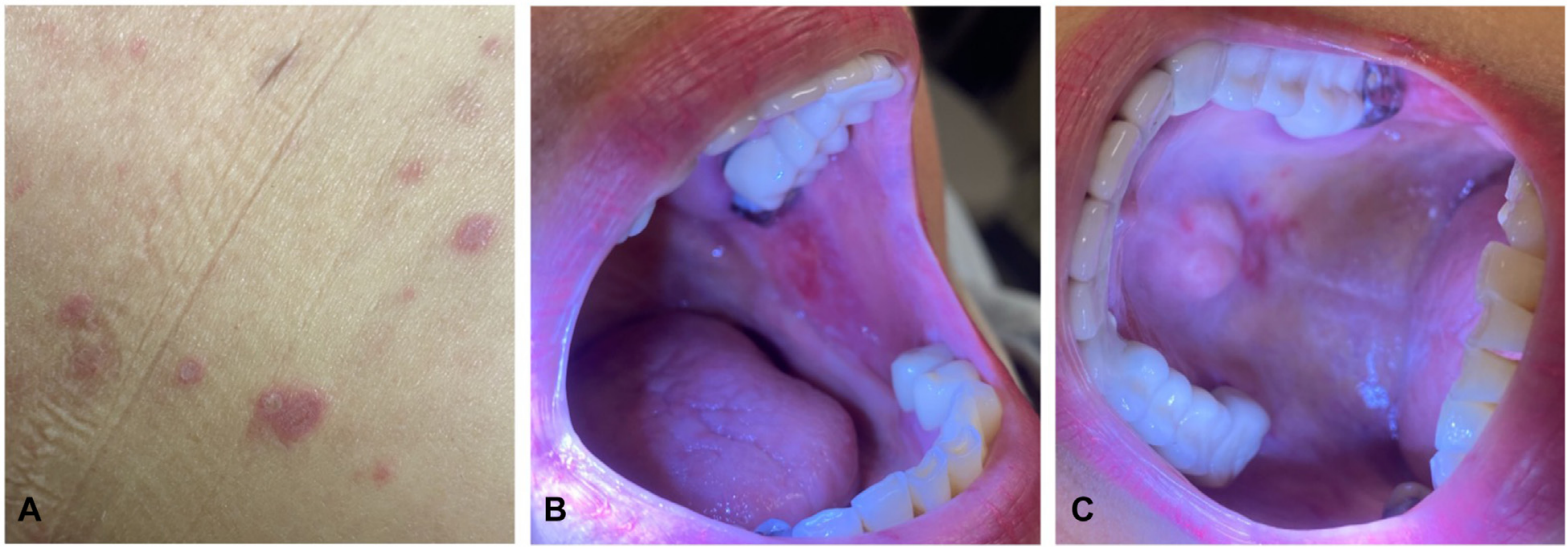
Clinical images of lichenoid eruption at presentation. **A,** Violaceous flat-topped papules and bullae on the right lateral aspect of the lower portion of the abdomen. **B,** Multiple lacy reticular erosions on the buccal mucosa and (**C**) hard palate consistent with erosive oral lichenoid eruption.

**Fig 2:**
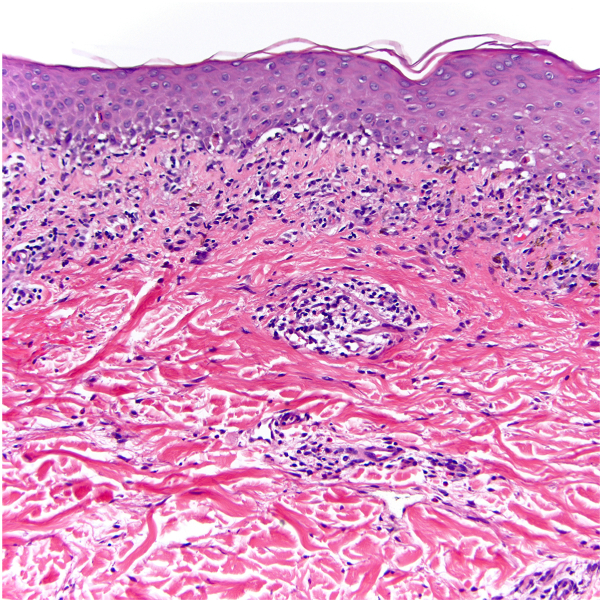
Lichenoid interface dermatitis with papillary to focally reticular dermal perivascular lymphocytic infiltrate and admixed scattered eosinophils, consistent with a lichenoid drug reaction. (Hematoxylin-eosin stain; original magnification: ×200)

A 67-year-old woman with a history of metastatic endometrial carcinoma that had progressed after surgery, brachytherapy, and chemotherapy was started on pembrolizumab and lenvatinib. She presented to dermatology clinic 1 month after her first cycle of treatment with painful oral sores as well as a progressive pruritic rash. Examination was notable for violaceous flat-topped papules, many with overlying bullae, over her lower extremities, abdomen, and back (Fig 1, *A*). Oral erosions were also noted on the hard palate and buccal mucosa with overlying lacy reticular Wickham striae (Fig 1, *B*, *C*). Pathology obtained from affected skin on the abdomen revealed a lichenoid interface dermatitis with a perivascular lymphoeosinophilic infiltrate and negative direct immunofluorescence (Fig 2). Hepatitis C antibody testing was negative. A thorough review of her medications did not reveal any additional culprits associated with lichenoid drug eruptions.

The patient was diagnosed with immunotherapy-associated bullous and oral erosive lichenoid dermatitis based on the clinical and histologic findings. Her mucocutaneous eruption had no response to topical corticosteroids, antihistamines, and baking soda rinses previously recommended by her oncology team. She was started on oral hydroxychloroquine 200 mg twice daily, tacrolimus swish and spit solution 3 times daily in the oral mucosa, and triamcinolone ointment was continued. Based on multidisciplinary discussion and patient preference, her ICI was continued without interruption.

**Fig 3:**
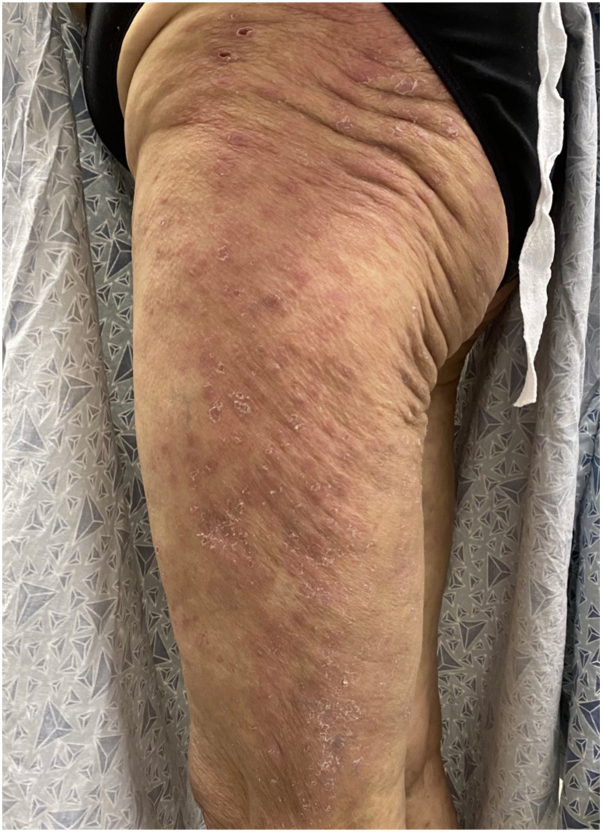
Violaceous papules coalescing into large plaques, with peripheral collarettes of scale and bullae over the left lateral aspect of the lower extremity, 1 month into hydroxychloroquine and tacrolimus treatment.

One week later the patient received another infusion of pembrolizumab leading to rash progression with more extensive violaceous plaques over the flank and extremities (Fig 3). Her rash had now become painful and was limiting activities of daily living. Her previously noted oral involvement had significantly improved with the tacrolimus solution.

**Fig 4:**
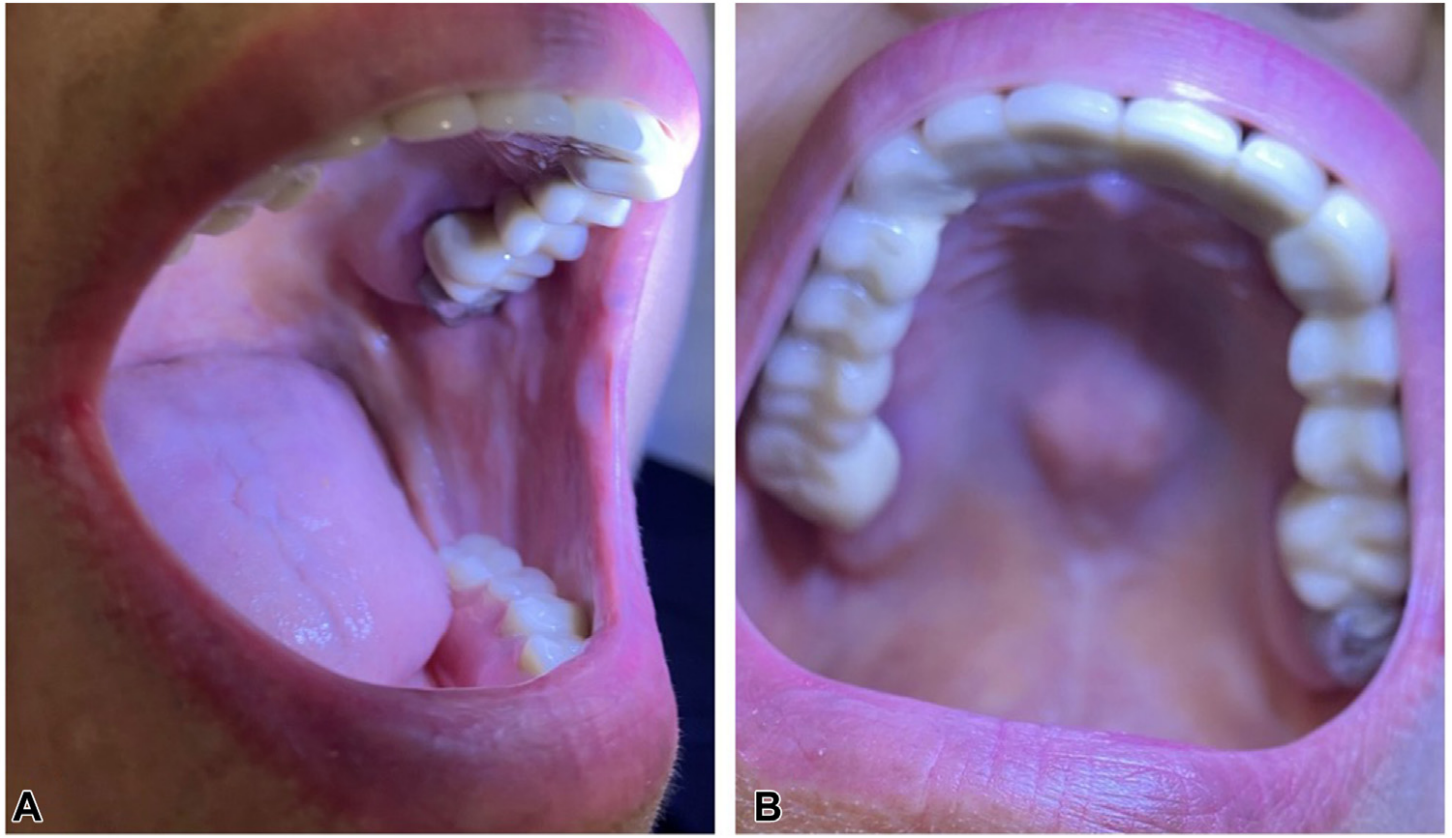
Clinical images of oral lichenoid eruption after treatment. **A,** Complete clearance of erosions on the buccal mucosa and (**B**) hard palate, 6 months into hydroxychloroquine treatment.

Given progression of her lichenoid eruption, pembrolizumab was temporarily held and a prednisone taper was started to allow time for hydroxychloroquine’s therapeutic effect. All other medications, including lenvatinib, remained uninterrupted. Two months after hydroxychloroquine initiation, the patient was tapered off prednisone entirely, discontinued topical steroids, and her ICI was resumed; at this time, she was entirely clear of both oral and skin disease (Fig 4, *A*, *B*). The patient tolerated resumption of pembrolizumab while on hydroxychloroquine with no recurrence of cutaneous or oral lichenoid eruption. She did not require any further systemic or topical corticosteroids.

Unfortunately, the patient was noted to have disease progression about 4 months later and was switched to targeted therapy; hydroxychloroquine was discontinued shortly after she came off immunotherapy without recurrence of eruption.

## Discussion

Our case highlights an immunotherapy-associated bullous and oral erosive lichenoid eruption successfully managed with maintenance hydroxychloroquine, allowing the patient to resume ICI for treatment of her malignancy despite an initially extensive mucocutaneous toxicity. It is imperative to identify maintenance therapies for lichenoid eruptions as they are one of the most common cutaneous toxicities of ICIs.^2^ Topical tacrolimus solution, which has demonstrated efficacy in cases of oral lichen planus and oral lichenoid eruptions, likely also contributed to resolution of our patient’s oral erosive involvement.^4^ Prednisone also contributed to improvement short-term, however on monotherapy with hydroxychloroquine the patient had sustained remission of skin disease despite ongoing ICI exposure.

Hydroxychloroquine downregulates major histocompatibility complex class II-peptide complex formation.^5^ This results in decreased activation of CD4^+^ T cells, diminishing the immune response, which may be the underlying driver of efficacy in lichenoid eruptions.^5^ Hydroxychloroquine also interferes with toll-like receptor signaling, decreasing proinflammatory cytokine production.^5^ Hydroxychloroquine is increasingly being used as a systemic agent to treat challenging lichenoid eruptions, with 1 multicenter retrospective study noting a favorable response in 84.4% of patients with lichen planus, an idiopathic analog to drug-induced lichenoid eruptions.^6^ Hydroxychloroquine has also been well-documented to improve erosive oral lichen planus in particular, relieving associated pain and reducing erythema in as quickly as 1 month.^4^ It is thus possible it contributed to our patient’s rapid resolution of oral involvement. Compared with traditional first-line agents such as corticosteroids, which cannot be used long-term, hydroxychloroquine is suggested to have lower recurrence rates of oral lichen planus, likely due to its extended treatment duration with a delayed onset.^7^^,^^8^

Other alternatives for lichenoid eruption control, such as acitretin and methotrexate, were considered but ultimately deemed less favorable. Methotrexate is a global immunosuppressant with numerous potential side effects, such as hepatotoxicity and cytopenias, which can also occur from other antineoplastic agents. Acitretin is associated with teratogenicity and should be avoided in women of childbearing age, as well as patients with regular alcohol intake.^3^ Hydroxychloroquine was thus selected as it is not globally immunosuppressive, with fewer adverse effects and minimal monitoring required aside from ophthalmologic screenings. However, there is scant literature available regarding hydroxychloroquine leading to resolution of cutaneous immune-related adverse effects, with only 2 case reports thus far on its use in ICI-induced lichenoid eruptions. Both cases reported beneficial patient outcomes and resolution of eruption without disease recurrence.^9^^,^^10^

In conclusion, systemic therapy with hydroxychloroquine led to sustained resolution of oral erosive and bullous lichenoid eruption in our case while also allowing for continuation of ICI treatment without recurrence of mucocutaneous toxicity. Hydroxychloroquine should be further studied as a promising systemic therapy for ICI-associated lichenoid eruptions given it requires less monitoring and has a more favorable side effect profile compared with traditional immunosuppressives. Additionally, any potential effects on ICI efficacy require further investigation.

## Conflicts of interest

None disclosed.
